# Differential effect of vinorelbine versus paclitaxel on ERK2 kinase activity during apoptosis in MCF-7 cells

**DOI:** 10.1054/bjoc.2001.2107

**Published:** 2001-09-01

**Authors:** X M Liu, L G Wang, W Kreis, D R Budman, L M Adams

**Affiliations:** Don Monti Division of Medical Oncology, North Shore University Hospital, NYU School of Medicine, Manhasset, New York, 11030 and; Glaxo Wellcome Inc., 5 Moore Drive, Research Triangle Park, NC, 27709, USA

**Keywords:** vinorelbine, MAP kinase, ERK2, apoptosis, bcl-2 phosphorylation, poly (ADP) ribose polymerase

## Abstract

The effects of vinorelbine and paclitaxel on the activity of extracellular signal-regulated protein kinase2 (ERK2), a member of MAP kinase, and its role in the induction of bcl-2 phosphorylation and apoptosis were evaluated in MCF-7 cells. We demonstrated that ERK2 was activated rapidly by vinorelbine, and was inhibited by either paclitaxel or estramustine. A 3-fold increase of ERK2 kinase activity was observed within 30 min when MCF-7 cells were treated with 0.1 μM vinorelbine. In contrast, the same treatment with paclitaxel resulted in a significant decrease of ERK2 kinase activity. We also demonstrated that elevated bcl-2 phosphorylation induced by vinorelbine is paralleled by decrease of a complex formation between bcl-2 and bax, cleavage of poly (ADP) ribose polymerase (PARP) protein, activation of caspase-7, and apoptosis. The levels of bcl-2 phosphorylation, bax, and PARP were not significantly affected by 2′-amino-3′-methoxyflavone (PD 98059), an ERK kinase specific inhibitor. Thus, our data suggest that the apoptosis induced by vinorelbine in MCF-7 cells is mediated through the bcl-2 phosphorylation/bax/caspases pathways, and that activation of ERK2 by vinorelbine does not directly lead to the drug-mediated apoptosis. Since decrease of PARP occurred quickly following the treatment of MCF-7 cells with either 0.1 μM of vinorelbine or paclitaxel, this protein may serve as an early indicator of apoptosis induced not only by DNA damaging agents, but also by antimicrotubule drugs.   http://www.bjcancer.com © 2001 Cancer Research Campaign


*British Journal of Cancer* (2001) **85**(9), 1403–1411
© 2001 Cancer Research Campaign
doi: 10.1054/ bjoc.2001.2107, available online at http://www.idealibrary.com on

### Differential effect of vinorelbine versus paclitaxel on ERK2 kinase activity during apoptosis in MCF-7 cells

**XM Liu[1], LG Wang[1], W Kreis[1], DR Budman[1] and LM Adams[2]**

[1]Don Monti Division of Medical Oncology, North Shore University Hospital, NYU School of Medicine, Manhasset, New York 11030 and [2]Glaxo Wellcome Inc., 5 Moore Drive, Research Triangle Park, NC 27709, USA

**Summary** The effects of vinorelbine and paclitaxel on the activity of extracellular signal-regulated protein kinase2 (ERK2), a member of MAP kinase, and its role in the induction of bcl-2 phosphorylation and apoptosis were evaluated in MCF-7 cells. We demonstrated that ERK2 was activated rapidly by vinorelbine, and was inhibited by either paclitaxel or estramustine. A 3-fold increase of ERK2 kinase activity was observed within 30 min when MCF-7 cells were treated with 0.1 μM vinorelbine. In contrast, the same treatment with paclitaxel resulted in a significant decrease of ERK2 kinase activity. We also demonstrated that elevated bcl-2 phosphorylation induced by vinorelbine is paralleled by decrease of a complex formation between bcl-2 and bax, cleavage of poly (ADP) ribose polymerase (PARP) protein, activation of caspase-7, and apoptosis. The levels of bcl-2 phosphorylation, bax, and PARP were not significantly affected by 2′-amino-3′-methoxyflavone (PD 98059), an ERK kinase specific inhibitor. Thus, our data suggest that the apoptosis induced by vinorelbine in MCF-7 cells is mediated through the bcl-2 phosphorylation/bax/caspases pathways, and that activation of ERK2 by vinorelbine does not directly lead to the drug-mediated apoptosis. Since decrease of PARP occurred quickly following the treatment of MCF-7 cells with either 0.1 μM of vinorelbine or paclitaxel, this protein may serve as an early indicator of apoptosis induced not only by DNA damaging agents, but also by antimicrotubule drugs. © 2001 Cancer Research Campaign http://www.bjcancer.com

**Keywords**: vinorelbine; MAP kinase; ERK2; apoptosis; bcl-2 phosphorylation; poly (ADP) ribose polymerase

The abbreviations used here are: ERK2: extracellular signal-regulated protein kinase 2; MAP kinase: mitogen-activated protein kinase; ERKs; extracellular signal-regulated protein kinase; PARP: poly (ADP) ribose polymerase; NBV: vinorelbine; PD 98059: 2′-amino-3′-methoxyflavone.

Apoptosis plays a crucial role in embryonic development and tissue homeostasis (White, 1996). Regulation of apoptosis involves a large number of genes that can be classified into three broad categories: (1) genes that primarily suppress apoptosis, such as bcl-2, bfl-1, brag-1, ced-9, BHRF-6; (2) genes that promote apoptosis such as bax, bak, bid, caspases family; and (3) genes upstream of apoptosis, including Fas/Fas ligand, p53, p63, myc, $P21^{WAF1/CIPI}$ (Boise et al, 1993; Henderson et al, 1993; Hengartner and Horvitz, 1994; Boyd et al, 1995). Apoptosis in mammalian cells is controlled by equilibrium between suppressor and promoter gene products (Oltvai and Korsmeyer 1994; Wang et al, 1999a). Bcl-2, the first identified anti-apoptotic gene product (Reed, 1994), plays an important role in the regulation of apoptosis. Although the functional significance of bcl-2 phosphorylation/dephosphorylation remains controversial, several reports have suggested that this post-translational modification may be a possible mechanism involved in the bcl-2 mediated apoptosis (Haldar et al, 1995; Haldar et al, 1997; Yamamoto et al, 1999).

Vinorelbine is a third-generation vinca alkaloid targeting microtubules with broad anticancer activity in man and is commercially available in many parts of the world (Budman, 1997). Our recent study showed synergism or antagonism with vinorelbine and paclitaxel were both sequence dependent and cell line-specific (Budman et al, 2000). In contrast with taxanes that are known to interact with polymerized tubulin and prevent depolymerization, vinca alkaloids interact with monomeric tubulin and prevent polymerization. Previous studies have demonstrated that these microtubule-targeting agents promote apoptosis in cancer cells (Donaldson et al, 1994; Haldar et al, 1995). A suggested mechanism of anti-cancer activity of paclitaxel has been loss of bcl-2 function as a consequence of phosphorylation by activated raf-1 kinase (Blagosklonny et al, 1995; Blagosklonny et al, 1996; Blagosklonny et al, 1997). MAP kinases also have been shown to be involved directly or indirectly in the regulation of bcl-2 phosphorylation (Avruch et al, 1994; Nishio et al, 1995; Lieu et al, 1998; Wang et al, 1998a; Attalla et al, 1998). In this study, we evaluated the effect of vinorelbine and paclitaxel on the activity of extracellular signal-regulated protein kinase2 (ERK2), a member of MAP kinase, and its possible role in the induction of bcl-2 phosphorylation and apoptosis in MCF-7 cells.

*Received 15 May 2001*
*Revised 14 August 2001*
*Accepted 15 August 2001*

*Correspondence to:* LG Wang, Mount Sinai School of Medicine, Division of Medical Oncology, One Gustave L. Levy Place, Box 1129, New York, NY 10029, USA, or DR Budman, Department of Medicine, North Shore University Hospital, 300 Community Drive, Mannhasset, NY 10030, USA

### MATERIALS AND METHODS

### Reagents

Antibodies against c-raf-1, ERK2, bcl-2, bax, caspase-7, and β-actin as well as anti-rabbit IgG-HRP were purchased from Santa Cruz Biotechnology, Inc. (Santa Cruz, CA). PARP antibody that only recognized original form (116 kDa) was purchased from Enzyme System Products (Livermore, CA), and PARP antibody that can recognize both original (116 kDa) and degradation form (83 kDa) was purchased from Boehringer Mannheim (Germany). Protein A and λ protein phosphatase (λPPase) was obtained from Calbiochem (San Diego, CA). A Western blotting detection kit was purchased from Amersham (Arlington Heights, IL). Reagents for SDS-PAGE and protein determination were obtained from Bio-Rad, (Richmond, CA). RPMI-1640 medium and fetal bovine serum (FBS) were purchased from Gibco BRL Life Technologies, Inc. (Gaithersburg, MD). Histone III-S (approximately 30 kDa), and myelin basic protein (18 kDa), as well as paclitaxel and other chemicals were purchased from the Sigma Chemical Company (St Louis, MO). $\gamma$-$^{32}$P-ATP and $^{32}$P-orthophosphate were purchased from NEN Life Science Products Inc. (Boston, MA). Vinorelbine (NVB) was kindly provided by Glaxo Wellcome Inc. (Research Triangle, NC).

### Cell culture

The breast cancer line MCF-7 was purchased from the American Type Culture Collection (Rockville, MD). Cells were maintained under conditions as described previously (Wang et al, 1996). The cell lines used for experiments were less than 30 passages in length.

### Cell treatment

MCF-7 cells at 65–75% confluence in RPMI-1640 medium containing 10% FBS were treated with indicated concentrations of vinorelbine or paclitaxel for indicated periods of time, or for 24 h. The cells were harvested and then washed with cold PBS. Total cellular proteins were extracted as described previously (Wang et al, 1994).

### Western blotting assay

Fifty to 100 μg of cellular extracts were separated by stack 4%/10% SDS-PAGE, or by 4/20% gradient SDS-PAGE, electro-transferred to nitrocellulose filters, and probed with bcl-2 or bax-specific antibody using standard techniques (Wang et al, 1994). Quantitation by densitometry of the ECL films was done using an Imaging Densitometer Model GS-700 (Bio-Rad Lab, Hercules, CA).

### Metabolic labeling and λ protein phosphatase treatment

MCF-7 cells grown exponentially were treated for 16 h with 0.2 μM of paclitaxel in phosphate-free RPMI-1640 medium containing 10% FBS, and metabolically labelled for 4 h by the addition of 200 μCi/ml of $^{32}$P-orthophosphate to the culture. The cells were harvested, washed and total proteins extracted with cell lysing buffer PBSTDS (0.058 M $NaHPO_4$, 0.017 M $NaHPO_4$, 0.068 M NaCl, 1% Trition X-100, 0.5% sodium deoxycholate and 0.1% sodium dodecyl sulfate) containing a cocktail of proteinase inhibitors (1 mM PSMF, 5 μg/ml leupeptin, 10 μg/ml aprotinin, and 25 μg/ml bacitracin) and phosphatase inhibitors (50 mM NaF and 1 mM sodium orthovanadate). The protein extracts were incubated with bcl-2 antibody at 4˚C for 1 h followed by addition of protein agarose A, and the immuno-reaction mixtures were incubated at 4˚C overnight with gentle rotation. After 4 washes with PBSTBS, the immunoprecipitates were resuspended in λPPase reaction buffer supplied by the manufacturer and incubated with 500 U of λPPase for 30 min at 30˚C in the presence or absence of phosphatase inhibitors (50 mM NaF, 2 mM sodium orthovanadate, 5 mM EDTA, and 5 mM EGTA). Protein sample buffer was then added, and after boiling for 2 min the reaction mixtures were subjected to Western blot analysis.

### Flow cytometry

MCF-7 cells at exponential growth were exposed to 0.1 μM of vinorelbine or paclitaxel for 48 h. The cells were harvested, washed with PBS and fixed with 70% ethanol for 1 h in ice. The cells were then treated with 500 μg of RNase for 30 min at 37˚C, stained with propidium iodide, and analysed by the FACSVantage (Becton, Dickinson and Company, Franklin Lakes, NJ) for cell cycle assay.

### ERK2 kinase assay

The ERK2 kinase assay was performed as previously described (Patton et al, 1998). Briefly, drug treated cells were harvested, washed once with ice-cold PBS and once with kinase buffer (20 mM HEPES, pH 7.4, 20 mM $MgCl_2$) in the presence of cocktail proteinase inhibitors (1 mM PSMF, 5 μg/ml leupeptin, 10 μg/ml aprotinin, and 25 μg/ml bacitracin) (Wang et al, 1995; Patton et al, 1998). Cells were lysed in PBSTDS (0.058 M $NaHPO_4$, 0.017 M $NaHPO_4$, 0.068 M NaCl, 1% Trition X-100, 0.5% sodium deoxycholate and 0.1% sodium dodecyl sulfate), and 100 μg of the proteins were immunoprecipitated overnight at 4˚C with antibody specifically against EKR2 in the presence of cocktail proteinase inhibitors. After three washes with PBSTDS and one wash with kinase buffer, the immuno-purified protein was incubated for 30 min in 50 μl kinase buffer containing 0.25 mg/ml of myelin and 100 μM/0.5 μCi [$\gamma$-$^{32}$P]-ATP at room temperature. The reaction was terminated by adding protein sample buffer and boiled for 1 min. Kinase activity was calculated from the amount of phosphorylated substrate formed using liquid scintillation counting as described previously (Sommercorn et al, 1987; Wang et al, 1995). Alternatively, an aliquot of the substrate was subjected to 4%/10% stack SDS-PAGE (Nishio et al, 1995), and the phosphorylated substrate was analyzed by autoradiography, and quantitated by an imaging densitometer.

### RESULTS

### Paclitaxel-mediated induction of bcl-2 phosphorylation

It has been previously demonstrated that paclitaxel stimulates bcl-2 phosphorylation in various cell lines (Yamamoto et al, 1999; Blagosklonny et al, 2000). To examine whether this is also the case

© 2001 Cancer Research Campaign for MCF-7 cells, we exposed MCF-7 cells to 0.2 μM of paclitaxel for 16 h in phosphate-free RPMI-1640 medium containing 10% FBS. The cells were then metabolically labeled with $^{32}$P-orthophosphate at 200 μCi/ml for 4 h. Immunoprecipitation of cell extracts from treated and untreated cells was performed in the presence or absence of λ protein phosphatase (λPPase) ± phosphatase inhibitors. As shown in Figure 1, treatment of paclitaxel significantly increased upper protein bands that were diminished by incubation with λPPase (lanes 2, and 3). Addition of phosphatase inhibitors (50 mM NaF, 2 mM of sodium orthovanadate, 5 mM EDTA, and 5 mM EGTA) to the reaction mixture reversed those dephosphorylated proteins (lane 4). These data were consistent with the previous report (Yamamoto et al, 1999), and demonstrated that like other cell lines, paclitaxel is also able to induce bcl-2 phosphorylation in MCF-7 cells.

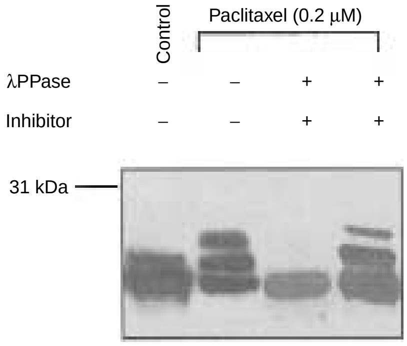

**Figure 1** Induction of bcl-2 phosphorylation by paclitaxel. MCF-7 cells grown exponentially were treated or untreated for 16 h with 0.2 μM of paclitaxel in phosphate-free RPMI 1640 medium containing 10% FBS. The cells were then metabolically labelled for 4 h by addition of 200 μCi/ml $^{32}$P-orthophosphate to the culture. The cells were harvested, washed and total proteins extracted for Western blot assay using bcl-2 antibody as described in 'Materials and Methods'. Lane 1, the immunoprecipitate was from untreated MCF-7 cells; Lane 2, from paclitaxel-treated cells; Lanes 3 and 4, the immunoprecipitates from paclitaxel-treated cells were resuspended in λPPase reaction buffer and incubated with 500 U of λPPase for 30 min at 30°C in the absence (lane 3) or presence (lane 4) of phosphatase inhibitors (50 mM NaF, 2 mM of sodium orthovanadate, 5 mM EDTA, and 5 mM EGTA)

### Induction of bcl-2 phosphorylation and bax expression

Exposure of MCF-7 cells grown exponentially to either 0.1 μM of vinorelbine or 0.1 μM of paclitaxel resulted in significant induction of bcl-2 phosphorylation (Figure 2A and Figure 3A). An approximately two-fold increase of bcl-2 phosphorylation, as reflected by the elevated ratio of phosphorylated-to-dephosphorylated bcl-2 proteins, occurred as early as 30 min following the treatment of MCF-7 cells with vinorelbine (Figure 2A). A hyper-phosphorylated bcl-2 band was detectable at 7 h, and significantly increased at 24 h. In contrast, the significant increase in bcl-2 phosphorylation by paclitaxel occurred at 7 h with a hyper-phosphorylated form of bcl-2 (Figure 1B). Similar induction of bcl-2 phosphorylation was also observed in cells treated with docetaxel (data not shown), but not with estramustine (Wang et al, 1999b).

The effects of vinorelbine or paclitaxel on the expression of bax were found to be time-dependent as illustrated in Figure 2B and Figure 3B. Both vinorelbine and paclitaxel at a concentration of 0.1 μM caused inhibition of bax expression in MCF-7 cells. A significant increase in the level of bax was observed at 24 h after the treatment with 0.1 μM of vinorelbine (Figure 2B). In contrast, the reduced level of bax in paclitaxel-treated cells recovered beginning at 3 h, and returned to the normal level at 7 h (Figure 3B).

Induction of bcl-2 phosphorylation is followed by loss of its ability to form heterodimers with bax (Haldar et al, 1996). Therefore, the complex formation between bcl-2 and bax was examined. As shown in Figure 4, vinorelbine significantly

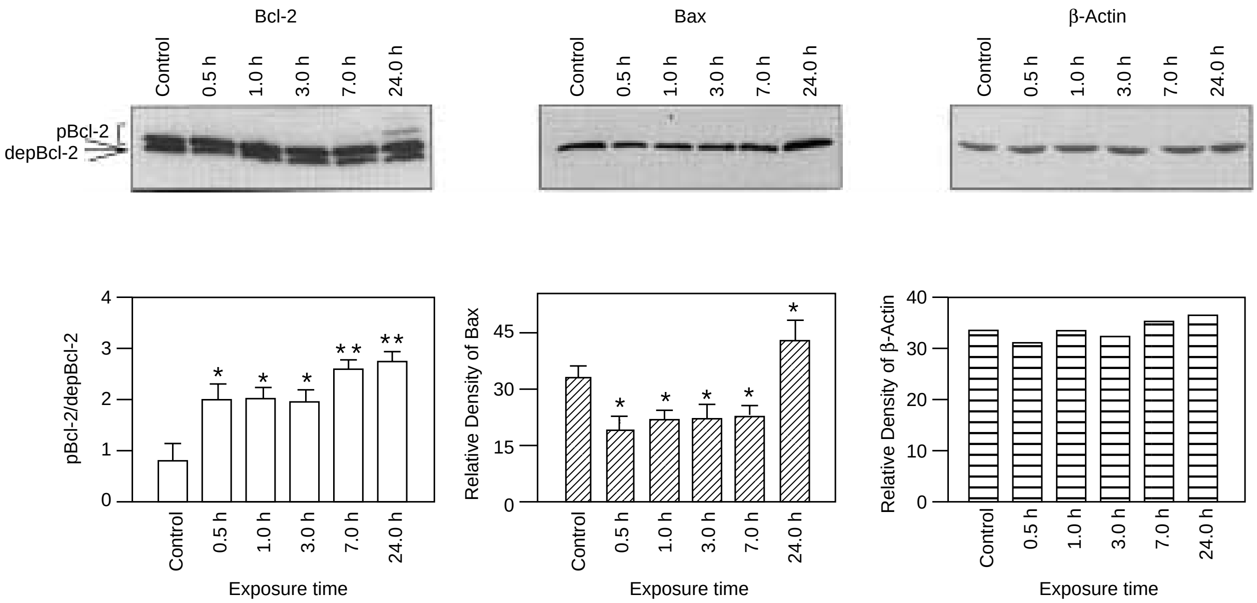

**Figure 2** Induction of bcl-2 phosphorylation in MCF-7 cells by vinorelbine. MCF-7 cells grown exponentially were exposed to 0.1 μM of vinorelbine for indicated periods of time. The cells were harvested, washed, and total protein extracted. Fifty μg proteins were subjected to 4%/20% gradient SDS-PAGE followed by Western blotting using specific antibodies against bcl-2, and bax, respectively (upper panel). The same membrane was stripped and re-probed with β-actin for loading control. The levels of bcl-2 and bax were measured from ECL film by Model GS-700 Imaging Densitometer (lower panels) and normalized by β-actin. *: $P < 0.05$; **: $P < 0.01$

© 2001 Cancer Research Campaign

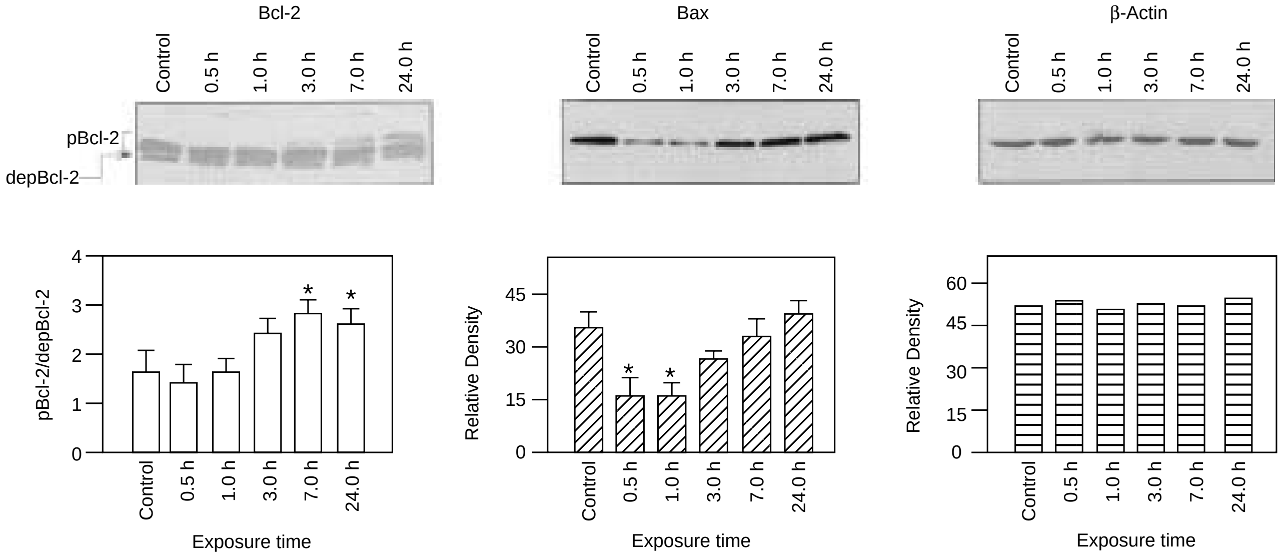

**Figure 3** Effect of paclitaxel on apoptotic related protein expression/phosphorylation in MCF-7 cells. MCF-7 cells at exponential growth were treated with 0.1 μM of paclitaxel for indicated periods of time. The cells were harvested, washed, and total protein extracted. Fifty μg of proteins were separated by 4%/20% gradient SDS-PAGE and electro-transferred to nitrocellulose membrane. Bcl-2 and bax proteins were then immuno-detected by Western blotting using specific antibodies (upper panel). The same membrane was stripped and re-probed with β-actin for loading control. The levels of bcl-2 and bax were measured from ECL film by Model GS-700 Imaging Densitometer and normalized by β-actin (lower panels). *: $P < 0.05$; **: $P < 0.01$

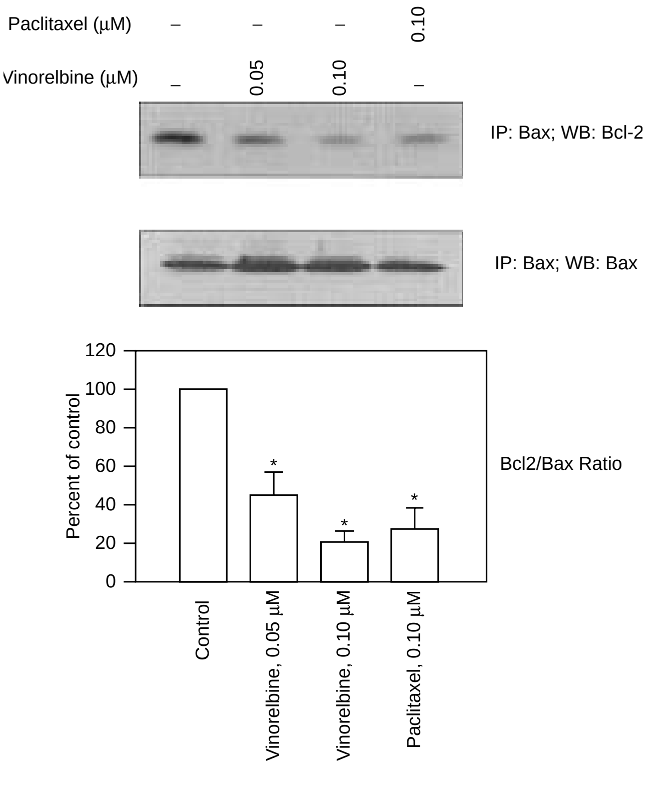

**Figure 4** Inhibition of the complex formation between bcl-2 and bax by vinorelbine in MCF-7 cells. MCF-7 cells, grown exponentially, were treated with indicated concentrations of vinorelbine for 24 h. The cells were harvested, washed, and proteins extracted. One hundred μg of proteins were immunoprecipitated with bax-specific antibody at 4˚C overnight in the presence of protein agarose A + G. The precipitates were subjected to Western blotting assay using specific antibodies against either bcl-2 and or Bax (upper panel). The ratio of bcl-2/bax was calculated from density of ECL film obtained by Model GS-700 Imaging Densitometer (lower panel). *: $P < 0.01$

decreased formation of bcl-2/bax heterodimers after the induction of bcl-2 phosphorylation, even though the level of bax protein was increased at that time (24 h). A similar observation has been reported in paclitaxel-, vincristine-, and vinblastine-treated cells (Haldar et al, 1996; Srivastava et al, 1998).

### Induction of apoptosis by vinorelbine and paclitaxel

In contrast to the finding of elevated bcl-2 phosphorylation, subsequent to exposure of MCF-7 cells to either 0.1 μM of vinorelbine or paclitaxel, the level of PARP (116 kDa) decreased significantly and rapidly. Approximately 44% decrease of PARP protein occurred within 30 min after the exposure, and a 73% decrease at 7 h, which was maintained at lower levels for up to 24 h (Figure 5A). The decreased PARP p116 paralleled its increase of degradation form p83 (Figure 5B). Since MCF-7 has no caspase 3, but caspase 7 has been indicated to be involved in the paclitaxel-induced PARP degradation and apoptosis (Panvichian et al, 1999) we examined caspase 7 activity in MCF-7 cells exposed to either vinorelbine or paclitaxel. As expected, a p20 active form of casepase 7 activity in MCF-7 cells exposed to either vinorlbine or paclitaxel. As expected, a p20 active form of caspase-7 was observed only in drug-treated cells (Figure 5B), which paralleled DNA fragmentation as measured by DNA agarose electrophoresis (Figure 5B, lower panel).

In addition, a significant apoptotic induction and $G_2$+M arrest by both vinorelbine and paclitaxel was also observed in MCF-1 cells. As shown in Figure 5C, approximately 20% of the cells became apoptotic after 48 h exposure to 0.1 μM of either vinorelbine or paclitaxel.

*© 2001 Cancer Research Campaign*

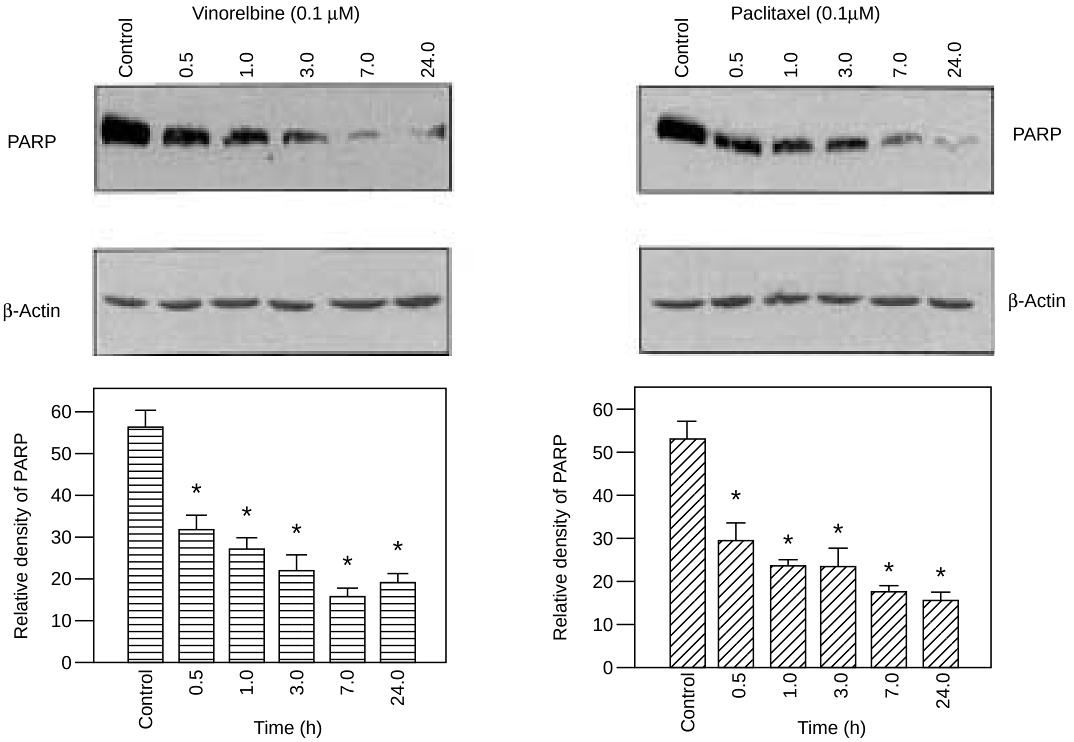

**Figure 5** Induction of apoptosis by vinorelbine and paclitaxel in MCF-7 cells. (**A**) Decrease in the level of PARP p116 following treatment with vinorelbine or paclitaxel. MCF-7 cells grown exponentially were exposed to 0.1 μM of vinorelbine or paclitaxel for indicated periods of time. The cells were harvested, washed, and total proteins extracted for Western blot analysis using antibodies against PARP or β-actin for loading control. The level of PARP was measured from ECL film by Model GS-700 Imaging Densitometer and normalized by β-actin (lower panels). *: $P < 0.05$; **: $P < 0.01$

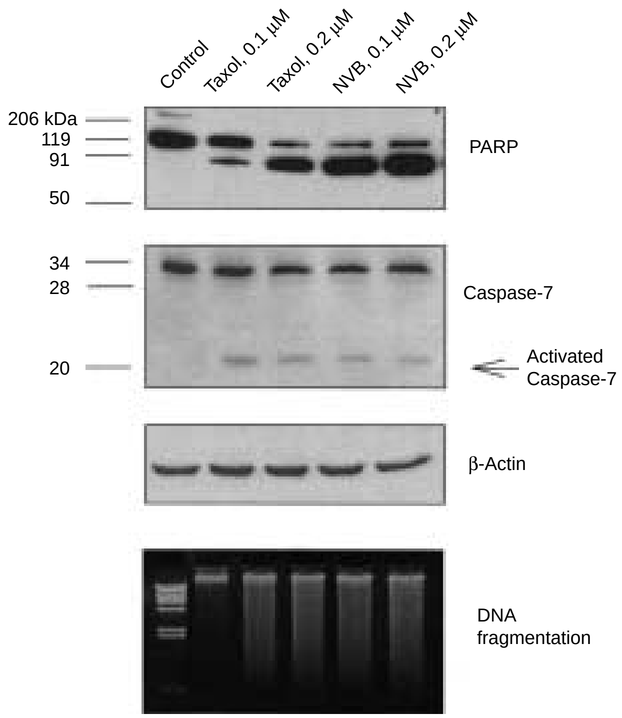

(**B**) Cleavage of PARA, activation of caspase-7 and DNA fragmentation induced by paclitaxel and vinorelbine. MCF-7 cells grown exponentially were treated for 24 h with indicated concentrations of either paclitaxel or vinorelbine. The cells were harvested, washed, and total proteins or DNA were extracted for determination of cleavage of PARA, or activation of caspase-7 by Western blot analysis or DNA fragmentation by DNA agarose gel electrophoresis, as described in 'Materials and Methods'

### Differential effect between vinorelbine and paclitaxel on ERK2 kinase activity

MAP kinase may be involved in the regulation of apoptosis (Wang et al, 1998b; Wang et al, 1999a), and may indirectly lead to bcl-2 phosphorylation (Avruch et al, 1994). To explore this possibility, the effects of exposure to either 0.1 μm vinorelbine or paclitaxel on KRK2 kinase activity were examined. As shown in Figure 5, ERK2 kinase activity, as represented by the levels of phosphorylated-myelin basic protein, was stimulated rapidly by vinorelbine (panel A), but was inhibited by paclitaxel (panel B) or estramustine (data not shown). An approximately 3-fold increase of KRK2 kinase activity was observed within 30 min in MCF-7 cells treated with 0.1μm vinorelbine. The maximal activation occurred at 1 h with a 4-fold increase, and the enzyme activity was maintained at higher levels for up to 24 h. In contrast, treatment with 0.1 μM paclitaxel resulted in a decrease of ERK2 kinase activity within 30 min, with significant inhibition (65%, $P < 0.05$) occurring at 1 h, followed by recovery of up to 7 h. Neither the activation nor inhibition of ERK2 kinase paralleled the increase in the level of phosphorylated bcl-2 induced by vinorelbine or paclitaxel, as shown in Figure 1.

No link between bcl-2 phosphorylation, bax expression, and ERK2 kinase activation induced by vinorelbine was demonstrated, as shown in Figure 6. No significant changes in the levels of either phosphorylated-bcl-2 or bax or PARP proteins were observed when cells were treated for 24 h with 0.1 μM of vinorelbine in the absence or presence of various concentrations (1.0–10.0 μM) of 2´-amino-3´-methoxyflavone, within which a concentration-dependent inhibition of PD 98059 on MAP kinase activity has been observed (data not shown) that was consistent with previous report (PD 98059, $IC_{50}$ = 2.0–7 μM (Alessi et al, 1995). In

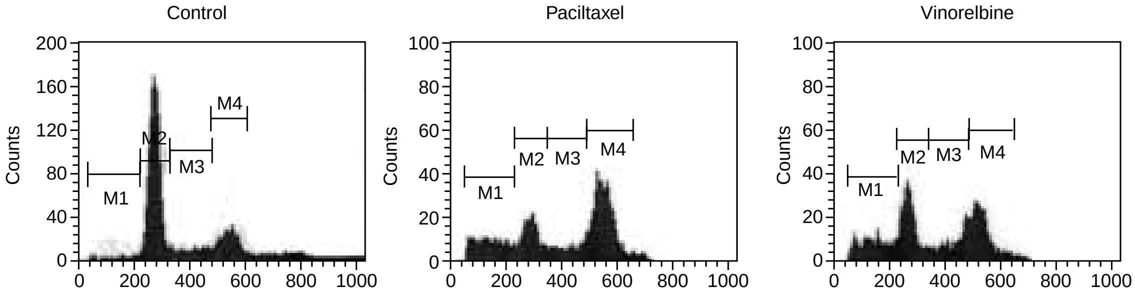

| | Control | Paciltaxel (0.1 µM) | Vinorelbine (0.1 µM) |
|---|---|---|---|
| Apoptotic cells (%) | 0.68 | 20.40 | 18.47 |
| $G_1$ | 70.87 | 20.76 | 18.47 |
| S | 7.95 | 10.22 | 16.01 |
| $G_2$+M | 17.71 | 48.21 | 32.36 |

**Figure 5(C)** Measurement of apoptotic cells by flow cytometry (FCM). MCF-7 cells at 70% confluent growth were exposed for 48 h to 0.1 µM of vinorelbine or paclitaxel. The cells were harvested, washed once with PBS, and fixed in 70% ethyl alcohol. The cells were then subjected to flow cytometry after treatment with RNase and IP staining, as described in 'Materials and Methods'

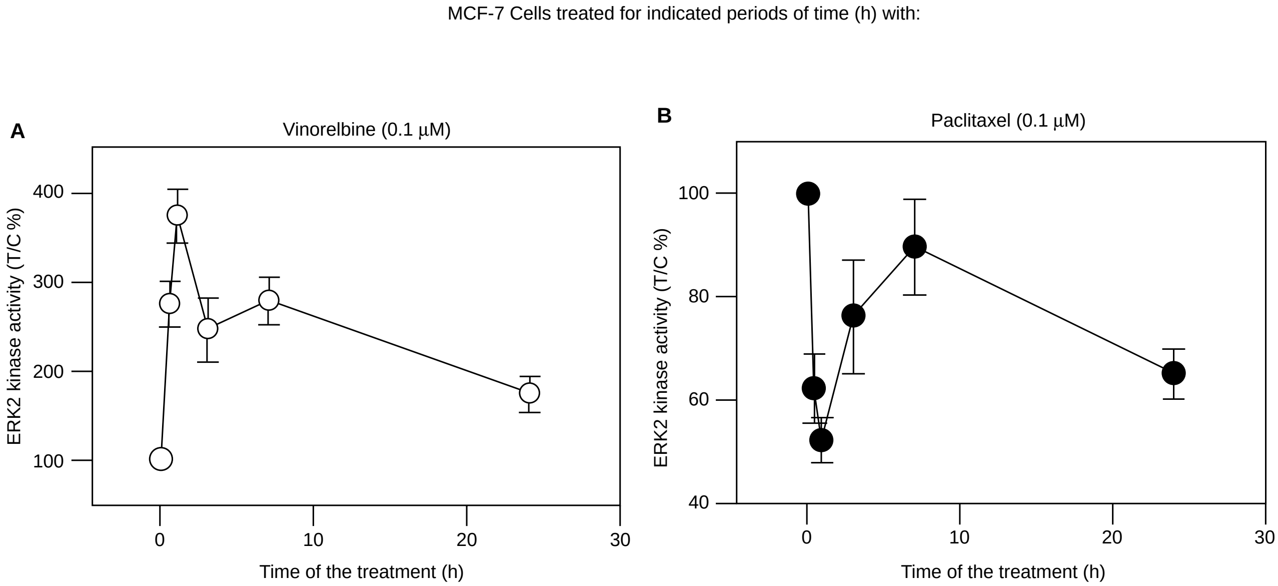

**Figure 6** Effect of vinorelbine and paclitaxel on ERK2 kinase activity. MCF-7 cells at exponential growth were treated with 0.1 µM vinorelbine (**A**) or with 0.1 µM paclitaxel (**B**) for indicated periods of time. The cells were harvested, washed, and proteins extracted. Immunopurified ERK2 kinase (using ERK2-specific antibody) was incubated in kinase buffer containing 5 µg myelin basic protein in the presence of 100 µM/0.5 µCi [$^{32}$P]-ATP for 10 min, and protein kinase activity was then determined using scintillation counting as described in 'Materials and Methods'. The data presents results of means ± SD obtained from three separate experiments addition, no phosphorylated bcl-2 protein bands were observed when immuno-purified bcl-2 was directly incubated with immuno-purified MAP kinase.

### DISCUSSION

Antimicrotubule agents, both polymerizing agents and depolymerizing drugs, have apoptosis-inducing activity (Donaldson et al, 1994) that is believed to result in the inactivation of bcl-2 function through phosphorylation (Haldar et al, 1995; Haldar et al, 1997; Srivastava et al, 1998) at serine-70 and serine-87 (Basu and Haldar 1998). Our study demonstrates that, analogous to paclitaxel, vinorelbine significantly and rapidly induces bcl-2 phosphorylation. The decrease of complex formation between bcl-2 and bax is also observed at 24 h after the exposure, even though the level of bax at that time is higher than that of untreated control, which is consistent with previous reports (Haldar et al, 1996; Srivastava et al, 1998).

Poly (ADP) ribose polymerase (PARP) is a chromatin-associated enzyme which, in response to DNA damage, binds rapidly to DNA strand breaks and undergoes automodification by forming long, branched poly (ADP-ribose) polymers (Sims et al, 1983; Lindahl et al, 1995). Although MCF-7 cells do not express caspase 3 that involve the cleavage of PARP (Janicke et al,

 *© 2001 Cancer Research Campaign*

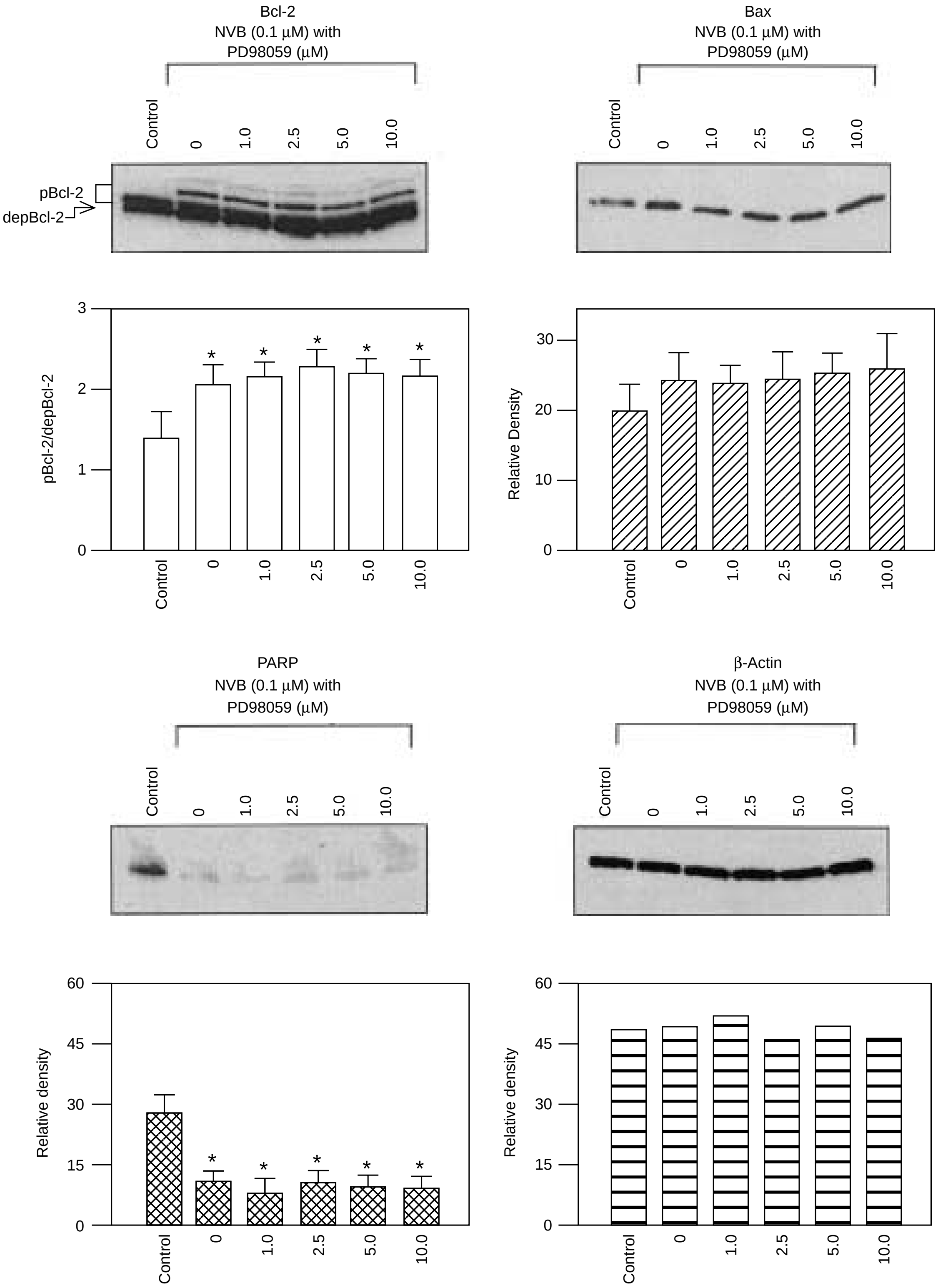

**Figure 7** Effects of MAP kinase inhibitor, PD98059, on the vinorelbine-mediated ERK2 kinase activation. MCF-7 cells grown exponentially were treated for 24 h with 0.1 μM of vinorelbine (NVB) in the presence of indicated concentrations of PD98059. The cells were harvested, washed, and total protein extracted for determination of levels of bcl-2, bax and PARP by Western blotting (upper panels), and quantitated by an Imaging densitometer (lower panel), and normalized by β-actin. The lower panel presents results of means ± SD obtained from three separate experiments. *: $P < 0.01$

1998), the selective cleavage of PARP by several other caspases is an important event during apoptosis since PARP cleavage and inactivation, as well as subsequent apoptotic process, are terminated by a peptide inhibitor of this protein (Sims et al, 1983; Kaufmann et al, 1993; Lazebnik et al, 1994; Lindahl et al, 1995; Tewari et al, 1995; Janicke et al, 1998; Germain et al, 1999; Rossini et al, 2001). In this study we found that the level of PARP p116 protein decreased rapidly and significantly following the treatment of MCF-7 cells with either vinorelbine or paclitaxel, which paralleled the activation of caspase 7 and DNA fragmentation. These data indicate that apoptosis induced by antimicrotubule drugs occurred very quickly, and that the activation of PARP protein not only serves as an early indicator of apoptosis mediated by DNA damage, but also by microtubule targeting agents.

MAP kinase subfamilies contain three members:

1. extracellular signal-regulated protein kinases (ERKs)
2. c-Jun N-terminal kinase/stress-activated protein kinase (JNK/SAPK)
3. p38 [Wang et al, 1998a]

Previous reports have suggested that JNK/SAPK may be responsible for bcl-2 phosphorylation (Avruch et al, 1994; Attalla et al, 1998; Yamamoto et al, 1999), and that microtubule targeting agents activate JNK/SAPK through both Ras and apoptosis signal-regulating kinase (ASK1) pathways in a variety of human cells. This activation requires interactions with microtubules (Wang et al, 1998a). The effects of antimicrotubule drugs on MAP kinase activity during apoptosis have also been reported (Lieu et al, 1998). In this study a different pattern of ERK2 activation in MCF-7 cells was observed, depending upon whether cells were exposed to vinorelbine or paclitaxel. Vinorelbine rapidly activated ERK2 kinase whereas paclitaxel inhibited the enzyme activity, a finding consistent with a previous report (Nishio et al, 1995). This process of activation/inactivation of ERK2 by the microtubule targeting agents is not consistent with the induction of bcl-2 phosphorylation by those drugs. No bcl-2 phosphorylation was observed when immuno-purified bcl-2 proteins were reacted directly with pure ERK2. Moreover, treatment of MCF-7 cells with PD 98059, a specific ERK2/p38 inhibitor (Alessi et al, 1995), did not block vinorelbine-induced bcl-2 phosphorylation. These data provide new evidence that ERK2 and p38 are not involved in antimicrotubule agent-mediated bcl-2 phosphorylation and apoptosis (Yamamoto et al, 1999), even though we cannot rule out the possibility of other MAP kinases being involved in vinorelbine-induced bcl-2 phosphorylation.

The functions of microtubule-associated proteins and filaments in microtubular assembly are regulated through their phosphorylation by MAP kinases and/or p34cdc2 (Raffaelli et al, 1992). Microtubule-associated proteins are good substrates for ERKs, and phosphorylation weakens their stabilizing effects on microtubule (Hirokawa, 1994). A differential effect of microtubule-targeting agents on MAP kinase activity during induction of apoptosis has been reported (Nishio et al, 1995). Previous studies have demonstrated that paclitaxel induces apoptosis through its inhibition of MAP kinase and p34cdc2 kinase activation at $G_2$/M phase in PC-9 and PC-14 cell lines (Nishio et al, 1995; Lieu et al, 1998). This decrease in MAP kinase and p34cdc2 kinase activities parallels the increased complex formation between microtubule-associated proteins with α- and β-tubulin, with an increase in the phosphorylation of microtubule-associated protein-2 (Nishio et al, 1995). Thus, activation or inactivation of microtubule targeting agents on ERK-2 kinase activity may depend upon whether the drug promotes polymerization or depolymerization. This effect may also be due to different binding sites of these drugs on microtubules, which then triggers distinct damage of microtubule structures and signal transduction pathways leading to apoptosis.

### ACKNOWLEDGEMENTS

The study was supported by the Don Monti Memorial Research Foundation, and Glaxo Welcome Inc.

### REFERENCES

Alessi DR, Cuenda A, Cohen P, Dudley DT and Saltil AR (1995) PD 098059 is a specific inhibitor of the activation of mitogen-activated protein kinase kinase in vitro and vivo. *J Biol Chem* **270**: 27489–27494

Attalla H, Westberg JA, Andersson LC, Adlercreutz H and Makela TP (1998) 2-Methoxyestradiol-induced phosphorylation of bcl-2: uncoupling from JNK/SAPK activation. *Biochem Biophys Res Commun* **247**: 616–619

Avruch J, Zhang X and Kyriakis JM (1994) Raf meets Ras: completing the framework of a signal transduction pathway. *Trends Biochem Sci* **19**: 279–283

Basu A and Haldar S (1998) Microtubule-damaging drugs triggered bcl-2 phosphorylation-requirement of phosphorylation on both serine-70 and serine-87 residues of bcl-2 protein. *Int J Oncol* **13**: 659–664

Blagosklonny MV, Schulte TW, Nguyen P, Mimnaugh EG, Trepel J and Neckers LM (1995) Taxol induction of p21WAF1 and p53 requires c-raf-1. *Cancer Res* **55**: 4623–4626

Blagosklonny MV, Schulte T, Nguyen P, Trepel J and Neckers LM (1996) Taxol-induced apoptosis and phosphorylation of bcl-2 protein involves c-raf-1 and represents a novel c-raf-1 signal transduction pathway. *Cancer Res* **56**: 1851–1854

Blagosklonny MV, Giannakakou P, el-Deiry WS, Kingston DG, Higgs PI, Necker LM and Fojo T (1997) Raf-1/bcl-2 phosphorylation: a step from microtubule damage to cell death. *Cancer Res* **57**: 130–135

Blagosklonny MV, Bishop PC, Robey R, Fojo T and Bates SE (2000) Loss of cell cycle control allows selective microtubule-active drug-induced Bcl-2 phosphorylation and cytotoxicity in autonomous cancer cells. *Cancer Res* **60**: 3425–3428

Boise LH, Gonzalez-Garcia M, Postema CE, Ding L, Lindsten T, Turka LA, Mao X, Nunez G and Thompson CB (1993) Bcl-x, a bcl-2-related gene that functions as a dominant regulator of apoptotic cell death. *Cell* **74**: 597–608

Boyd JM, Gallo GJ, Elangovan B, Houghton AB, Malstrom S, Avery BJ, Ebb RG, Subramanian T, Chittenden T and Lutz RT (1995) Bik, a novel death-inducing protein shares a distinct sequence motif with bcl-2 family proteins and interacts with viral and cellular survival promoting proteins. *Oncogene* **11**: 1921–1928

Budman DR (1997) Vinorelbine (Navelbine): a third-generation vinca alkaloid. *Cancer Invest* **15**: 475–490

Budman DR, Calabro A, Wang LG, Liu XM, Stiel L, Adams LM and Kreis W (2000) Synergism of cytotoxic effects of vinorelbine and paclitaxel in vitro. *Cancer Invest* **18**: 695–701

Donaldson KL, Goolsby GL, Kiener PA and Wahl AF (1994) Activation of p34cdc2 coincident with taxol-induced apoptosis. *Cell Growth Differ* **5**: 1041–1050

Germain M, Affar EB, D'Amours D, Dixit VM, Salvesen GS and Poirier GG (1999) Cleavage of automodified poly(ADP-ribose) polymerase during apoptosis. Evidence for involvement of caspase-7. *J Biol Chem* **274**: 28379–28384

Haldar S, Jena N and Croce CM (1995) Inactivation of bcl-2 by phosphorylation. *Proc Natl Acad Sci USA* **92**: 4507–4511

Haldar S, Chintapalli J and Croce CM (1996) Taxol induces bcl-2 phosphorylation and death of prostate cancer cells. *Cancer Res* **56**: 1253–1255

Haldar S, Basu A and Croce CM (1997) Bcl-2 is the guardian of microtubule integrity. *Cancer Res* **57**: 229–233

Henderson S, Huen D, Rowe M, Dawson C, Johnson G and Rickinoson A (1993) Epstein-Barr virus-coded BHRF1 protein, a viral homologue of Bcl-2, protects human B cells from programmed cell death. *Proc Natl Acad Sci USA* **90**: 8479–8483

Hengartner MO and Horvitz HR (1994) Celegans cell survival gene ced-9 encodes a functional homolog of the mammalian proto-oncogene bcl-2. *Cell* **76**: 665–676

Hirokawa N (1994) Microtubule organization and dynamics dependent on microtubule-associated proteins. *Curr Opin Cell Biol* **6**: 74–81

 *© 2001 Cancer Research Campaign*

Janicke RU, Sprengart ML, Wati MR and Porter AG (1998) Caspase-3 is required for DNA fragmentation and morphological changes associated with apoptosis. *J Biol Chem* **273**: 9357–9360

Kaufmann SH, Desnoyers S, Ottaviano Y, Davidson NE and Poirier GG (1993) Specific proteolytic cleavage of poly(ADP-ribose) polymerase: an early marker of chemotherapy-induced apoptosis. *Cancer Res* **53**: 3976–3985

Lazebnik YA, Kaufmann SH, Desnoyers S, Poirier GG and Earnshaw WC (1994) Cleavage of poly(ADP-ribose) polymerase by a proteinase with properties like ICE. *Nature* **371**: 346–347

Lieu CH, Liu CC, Yu TH, Chen KD, Chang YN and Lai YK (1998) Role of mitogen-activated protein kinase in taxol-induced apoptosis in human leukemic U937 cells. *Cell Growth Differ* **9**: 767–776

Lindahl T, Satoch MS, Poirier GG and Klungland A (1995) Post-translational modification of poly(ADP-ribose) polymerase induced by DNA strand breaks. *Trends Biochem Sci* **20**: 405–411

Nishio K, Arioka H, Ishida T, Fukumoto H, Kurokawa H, Sata M, Ohata M and Saijo N (1995) Enhanced interaction between tubulin and microtubule-associated protein 2 via inhibition of MAP kinase and CDC2 kinase by paclitaxel. *Int J Cancer* **63**: 688–693

Oltvai ZN and Korsmeyer SJ (1994) Checkpoints of dueling dimers foil death wishes. *Cell* **79**: 189–192

Panvichian R, Orth K, Pilat MJ, Day ML, Day KC, Yee C, Kamradt JM and Pienta KJ (1999) Signaling network of paclitaxel-induced apoptosis in the LNCaP prostate cancer cell line. *Urology* **54**: 746–752

Patton SE, Martin ML, Nelsen LL, Fang X, Mills GB, Bast RC Jr and Ostrowski MC (1998) Activation of the ras-mitogen-activated protein kinase pathway and phosphorylation of ets-2 at position threonine 72 in human ovarian cancer cell lines. *Cancer Res* **58**: 2253–2259

Raffaelli N, Yamauchi PS and Purichi DL (1992) Microtubule-associated protein auto-phosphorylation alters in vitro microtubule dynamic instability. *FEBS Lett* **296**: 21–24

Reed JC (1994) Bcl-2 and the regulation of programmed cell death. *J Cell Biol* **124**: 1–6

Rossini GP, Sgarbi N and Malaguti C (2001) The toxic responses induced by okadaic acid involve processing of multiple caspase isoforms. *Toxicon* **39**: 763–770

Sims JL, Berger SJ and Berger NA (1983) Poly(ADP-ribose) polymerase inhibitors preserve nicotinamide adenine dinucleotide and adenosine 5′′triphosphate pools in DNA damaged cells: mechanism of stimulation of unscheduled DNA synthesis. *Biochemistry* **22**: 5188–5194

Sommercorn J, Mulligan JA, Lozeman FJ and Krebs EG (1987) Activation of casein kinase II in response to insulin and to epidermal growth factor. *Proc Natl Acad Sci USA* **84**: 8834–8838

Srivastava RK, Srivastava AR, Korsmeyer SJ, Nesterova M, Cho-Chung YS and Longo DL (1998) Involvement of microtubules in the regulation of bcl-2 phosphorylation and apoptosis through cyclic AMP-dependent protein kinase. *Mol Cell Biol* **18**: 3509–3517

Tewari M, Quan LT, O'Rourke K, Desnoyers S, Zeng Z, Beidler DR, Poirier GG, Salvesen GS and Dixit VM (1995) Yama/CPP32b, a mammalian homolog of CED-3, is a crm-A-inhibitable protease that cleaves the death substrate poly(ADP-ribose) polymerase. *Cell* **81**: 801–809

Wang LG, Liu XM, Li ZR, Denstman S and Bloch A (1994) Differential binding of nuclear c-ets-1 protein to an intron I fragment of the c-myb gene in growth versus differentiation. *Cell Growth Differ* **5**: 1243–1251

Wang LG, Liu XM, Wikiel H and Bloch A (1995) Activation of casein kinase II in ML-1 human myeloblastic leukemia cells requires IGF-1 and transferrin. *J Leukocyte Biol* **57**: 332–334

Wang LG, Liu XM, Kreis W and Budman DR (1996) Involvement of prostate-specific antigen in the stimulation of LNCaP cell growth. *Oncol Report* **3**: 911–917

Wang TH, Wang HS, Ichijo H, Giannakakou P, Foster JS, Fojo T and Wimalasena J (1998a) Microtubule-interfering agents activate c-Jun N-terminal kinase/stress-activated protein kinase through both Ras and apoptosis signal-regulating kinase pathways. *J Biol Chem* **273**: 4928–4936

Wang X, Martindale JL, Liu Y and Holbrook NJ (1998b) The cellular response to oxidative stress: influences of mitogen-activated protein kinase signaling pathways on cell survival. *Biochem J* **333**: 291–300

Wang LG, Liu XM, Kreis W and Budman DR (1999a) The effect of antimicrotubule agents on signal transduction pathways of apoptosis: A review. *Cancer Chemother Pharmacol* **44**: 355–361

Wang LG, Liu XM, Kreis W and Budman DR (1999b) Synergistic effect of Estramustine and PSC 833 on the inhibition of androgen receptor phosphorylation. *Biochem Pharmacol* **58**: 1115–1121

White E (1996) Life, death and the pursuit of apoptosis. *Genes Dev* **10**: 1–15

Yamamoto K, Ichijo H and Korsmeyer SJ (1999) Bcl-2 is phosphorylated and inactivated by an ASK1/Jun N-terminal protein kinase pathway normally activated at G2/M. *Mol Cell Biol* **19**: 8469–8478

© 2001 Cancer Research Campaign 
